# Irreducible Lisfranc injury by tibialis anterior tendon entrapment: A case report

**DOI:** 10.1097/MD.0000000000024822

**Published:** 2021-03-19

**Authors:** Do-Yeon Kim, Jong-Kil Kim, Min-Woo Kim, Kwang Bok Lee

**Affiliations:** a Yeon Orthopaedic Clinic; b Department of Orthopaedic Surgery, Presbyterian Medical Center; c Department of Orthopedic Surgery, Jeonbuk National University Medical School, Research Institute of Clinical Medicine of Jeonbuk National University-Biomedical Research Institute of Jeonbuk National University Hospital, Jeonju, South Korea.

**Keywords:** entrapment, irreducible Lisfranc injury, tibialis anterior tendon

## Abstract

**Rationale:**

Lisfranc injuries are a dislocation of the metatarsal bones from the tarsal bone. Although closed reduction is possible in most cases of Lisfranc injury when attempted in the early stage, there are some rare cases for which open reduction is required. Herein we report a case of irreducible Lisfranc injury in a 34-year-old man who presented to our institution with painful swelling.

**Patient concerns:**

We report a 34-year-old man presented to our institution with painful swelling after a fall from 1.0 m height.

**Diagnoses:**

We diagnosed it as irreducible Lisfranc injury by tibialis anterior tendon entrapment through plain radiologic study and surgical findings.

**Interventions:**

Plain X-ray, C-arm fluoroscopy and open surgery were performed.

**Outcomes:**

We did a closed reduction under a C-arm fluoroscopic guide, but it was not successful. Thus, we had to do an open reduction of a Lisfranc dislocation. Upon exposure, we observed the entrapment of the tibialis anterior tendon between the medial and intermediate cuneiform bones.

**Lessons:**

Our report is valuable in that it can contribute to the diagnosis and suggest a clue to the treatment of such a rare pathology. The knowledge in the rare case of entrapment of the tibialis tendon and the understanding of management will be useful when a irreducible Lisfranc dislocation is unsuccessful after an attempt at closed reduction.

## 1 Introduction

Lisfranc injuries involve the displacement (or dislocation) of the metatarsal bones from the tarsus, particularly for the second tarsometatarsal joint and the Lisfranc ligament.^[1]^ They are also called Lisfranc fracture-dislocations, where there is a disruption in the articulation between the base of the second metatarsal bone and the medial cuneiform bone. Lisfranc injuries are caused when excessive kinetic energy is applied, either directly or indirectly, to the midfoot and are often seen in traffic collisions or industrial accidents.^[2]^ Progressive pain, deformity and chronic loss of function are often seen when immediate and adequate diagnosis and treatment are not applied. Anatomic reduction and stable fixation are necessary for the best results: displacement of more than 2 mm or gross instability of the tarsometatarsal, intercuneiform, or naviculocuneiform joints are unacceptable.^[3]^ The goal of the treatment is to obtain and maintain anatomic reduction and stability.^[3–7]^

Open reduction and rigid internal fixation are indicated for a displacement of more than 2 mm or any evidence of instability. Although closed reduction is possible in most cases of Lisfranc injury when attempted in the early stage, there are some rare cases for which open reduction is required. Herein we report a case of irreducible Lisfranc injury.

## 2 Consent

The patient signed informed consent for the publication of this case report and any accompanying images. Ethical approval of this study was waived by the ethics committee of Jeonbuk National University Hospital because it was a case report.

## 3 Case report

**Figure 1:**
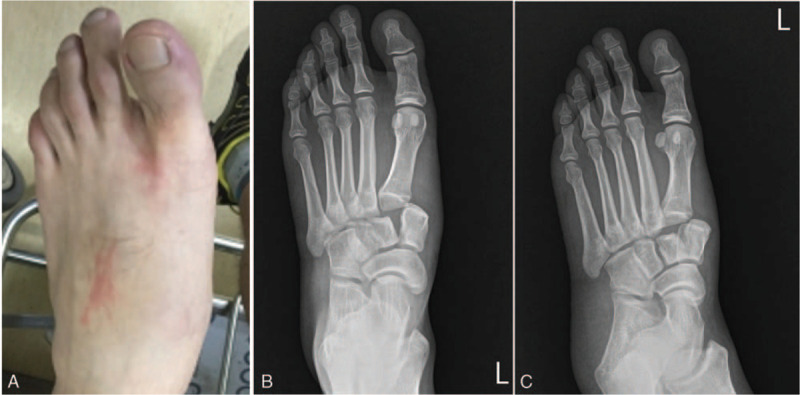
Preoperative photograph of patient's left foot, showing swelling with bruise and “toe up” sign (A). The foot anteroposterior (B) and medial oblique (C) plain radiograms showed partial incongruity of the tarsometatarsal joint with lateral dislocation.

A 34-year-old man presented to our emergency department after a fall from 1.0 m height. He was initially diagnosed with first Lisfranc dislocation and manual reduction was attempted at the private orthopedic clinic, but it was unsuccessful. His left foot was swollen and tender, with a slight “toe up” sign (Fig. 1-A). A plain radiogram of the foot showed partial incongruity of the tarsometatarsal joint with lateral dislocation (type B2, the Myerson Classification of the Lisfranc Injuries) (Fig. 1-B and C).

**Figure 2:**
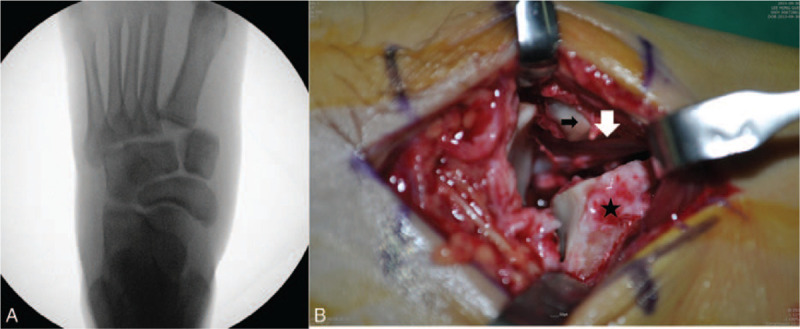
Failure of manual reduction under a C-arm fluoroscopic guide (A). Intra-operative photograph (B) showing the entrapment of the tibialis anterior tendon between the medial and lateral cuneiform bones.

We prepared for the emergency operation under spinal anesthesia and made several attempts at closed reduction under a C-arm fluoroscopic guide; these were also not successful (Fig. 2-A). Thus, we had no choice but to open for a reduction of the Lisfranc dislocation. A 5-cm incision was made between the first and second metatarsal bones; dislocated first and second rays were exposed, showing the entrapment of the tibialis anterior tendon between the medial and intermediate cuneiform bones (Fig. 2-B).

**Figure 3:**
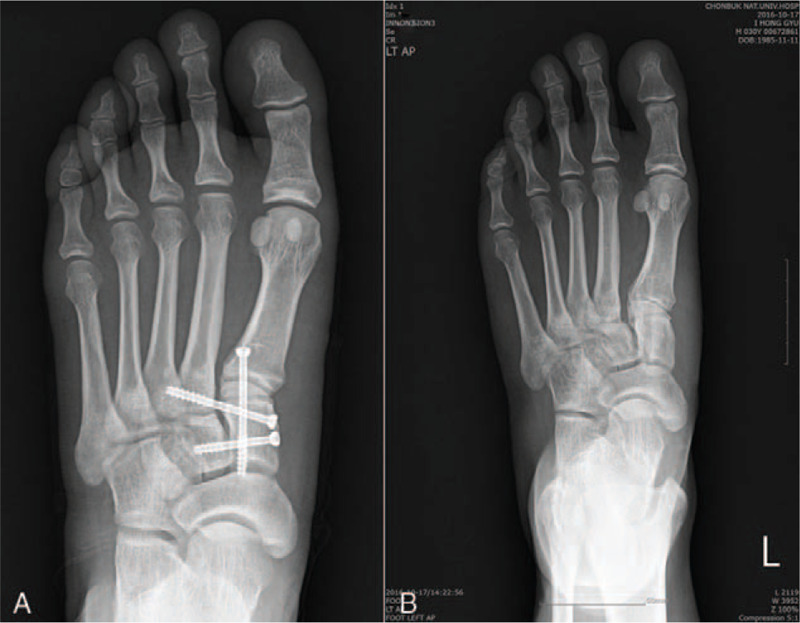
Postoperative (A) and 1-year follow-up (B) foot anteroposterior plain radiograms showing good reduction and no arthritic change in the Lisfranc joint.

The entrapped tibialis anterior tendon was released and repositioned to its anatomically normal position. Reduced segments of the base of the first metatarsal bone and the medial cuneiform bone, the medial and middle cuneiform bones, and the medial cuneiform bone and the base of the second metatarsal bone were rigidly fixed using 4.0-mm cannulated screws. The ruptured Lisfranc ligaments and intercuneiform ligaments were repaired with nonabsorbable sutures (ETHIBOND EXCEL Suture). Postoperative foot anteroposterior plain radiogram shows good reduction and fixation with screws in Lisfranc joint (Fig. 3-A). Stability was maintained in a postoperative instability test.

For his postoperative care, a short leg splint was applied until postoperative 2 weeks for immobilization. After that period, he wore an air cast boot without weight bearing until postoperative 8 weeks, and ankle ROM was recommended during this period. A gradual advance to full weight bearing was initiated at postoperative 8 weeks. Hardware was removed at postoperative one year (Fig. 3-B), and he returned to normal athletic activity at that time.

## 4 Discussion

The surgical treatment for the first Lisfranc dislocation includes CRIF (Closed Reduction and Internal Fixation) and ORIF (Open Reduction and Internal Fixation), both of which require long-term rehabilitation. The risk of posttraumatic OA will depend on the quality of the reduction.^[8]^ If not properly treated, it can result in chronic pain, planovalgus deformity, and severe loss of function.

The first choice for this patient was a CRIF (Closed Reduction and Internal Fixation) with percutaneous Kirschner-wire fixation, but we could not achieve anatomical reduction under spinal anesthesia in the operating room. We had to do an ORIF (Open Reduction and Internal Fixation) as an alternative choice.

What can be indicated for an ORIF (Open Reduction and Internal Fixation) to reduce a first Lisfranc dislocation? We learned from this case that the tibialis anterior tendon can be trapped between the medial and intermediate cuneiform bones in the event of a first Lisfranc dislocation. We found out we had to carry out an ORIF (Open Reduction and Internal Fixation) unless we achieved immediate anatomical reduction.

Not many cases have been reported about an irreducible Lisfranc dislocation so far, and the mechanism that hinders closed reduction has been reported as listed below:

1. Tendon interposition of either the tibialis anterior or the peroneus longus tendon.
2. Incongruity of the medial cuneiform bone and the first metatarsal articulation.
3. Interposition of a fracture fragment in the second metatarsal and the intermediate cuneiform joint

Only 8 cases have been reported since 1950 that are related to interposition of the tibialis anterior tendon.^[3–10]^ All the 9 cases, including this case, accounted for lateral dislocations of the tarsometatarsal joint according to the Myerson Classification. Eight cases of the 9 showed interposition of the tibialis anterior tendon between the medial and intermediate cuneiform bones, and only one case showed a position between the fractured parts of the second metatarsal bones.^[3–10]^

Denton reported that with laterally displaced metatarsals, the lateral slip of the anterior tibialis tendon can become interposed between the medial and intermediate cuneiforms, preventing anatomical reduction.^[9]^ There was a previous report suggesting that the “toe up” sign can predict an interposition of the tibialis anterior tendon that inhibits closed manual reduction. The lateral slip of the anterior tibia1 tendon inserting into the first metatarsal becomes trapped beneath the medial cuneiform. This insertion point then acts as a pivot, so that the dorsally applied reduction force on the base of the metatarsal causes the metatarsal to dorsiflex.^[10]^ Karaindros et al suggested that the surgeons should use CRIF for the treatment of Lisfranc fracture dislocations. Not all Lisfranc dislocations can be treated by CRIF, but no criteria exist about which should be treated initially by CRIF and which by ORIF. We indicate that a lateral Type A dislocation with an opening between the medial and the middle cuneiform is highly indicative of Tibialis Anterior interposition and therefore should be treated by ORIF. If CRIF is attempted but closed reduction is unsuccessful, then the surgeon should be aware of the possibility of tendon or bone fragment interposition.^[3]^

Only 8 cases have been reported in the literature about the entrapment of the tibialis anterior tendon accompanying first Lisfranc dislocation. Our report is valuable in that it can contribute to diagnosis and suggest a clue to the treatment of such a rare pathology. There are not enough cases or data to suggest a definite guideline for this rare injury, but there are some signs and tips that the surgeons should keep in mind and not hesitate to do an ORIF in case of an irreducible Lisfranc injury. In this patient, preoperative radiological evaluation with foot MRI was done. The MRI suggested the interposition of the tibialis anterior tendon between the medial and lateral cuneiform bones. Although distinct features and characteristics can be collected from our previous report, they have some limitations and lack accuracy for the exact diagnosis. We should resort to preoperative MRI for the exact preoperative diagnosis and surgical planning. When needing to do surgery without preoperative MRI, if the surgeon encounters a situation where the initial attempt with closed reduction fails, we suggest the surgeons should do an open reduction. The surgeon may expect the tibialis anterior tendon to be entrapped between the medial and intermediate cuneiform bones, which was the most common site, as previous cases have reported.

The knowledge about the rare case of entrapment of the tibialis tendon and the understanding of management will be useful in the situation where an irreducible Lisfranc dislocation cannot be successfully treated by closed reduction.

## Author contributions

**Conceptualization:** Jong-Kil Kim, Kwang-Bok Lee.

**Data curation:** Do-Yeon Kim, Jong-Kil Kim, Min-Woo Kim, Kwang-Bok Lee.

**Funding acquisition:** Kwang-Bok Lee.

**Investigation:** Min-Woo Kim.

**Methodology:** Jong-Kil Kim.

**Supervision:** Kwang-Bok Lee.

**Writing – original draft:** Do-Yeon Kim.

**Writing – review & editing:** Kwang-Bok Lee.
